# From Thermosensing to Reproduction: Plant Responses to Elevated Temperature

**DOI:** 10.3390/ijms27219840

**Published:** 2026-11-03

**Authors:** Fanglei He, Weiliang Chen, Jingyu Wu, Chao Fang, Shichen Li, Lingshuang Wang

**Affiliations:** Guangdong Provincial Key Laboratory of Plant Adaptation and Molecular Design, Guangzhou Key Laboratory of Crop Gene Editing, Innovative Center of Molecular Genetics and Evolution, School of Life Sciences, Guangzhou University, Guangzhou 510006, China

**Keywords:** thermosensing, seed germination, plant architecture, flowering, reproductive development

## Abstract

High temperatures threaten plant productivity and survival worldwide. Plants respond to warming across their life cycle, but the effects of elevated temperature depend on its intensity, duration, and rate of change, and the developmental and environmental context in which it is experienced. Here, we synthesize current knowledge of temperature sensing and developmental responses from seed germination through plant architectural acclimation, floral transition, and reproduction. We distinguish thermomorphogenesis under moderately elevated ambient temperatures from heat-stress responses that protect cellular and developmental integrity under more severe conditions, while emphasizing their mechanistic overlap. Across these stages, we highlight how temperature responses are shaped by organ identity, species, genotype, and photoperiod, and discuss the trade-offs that must be resolved to translate developmental plasticity into heat-resilient crop production.

## 1. Introduction

Rising temperatures increasingly threaten plant growth, biodiversity, and agricultural production. Human-driven climate change has increased the risk of high-temperature events, which can alter plant distribution and phenology and may endanger species with limited heat tolerance [1,2,3,4,5,6,7]. However, plants do not respond uniformly to all elevated temperatures. Mild warming typically induces thermomorphogenesis, an adaptive developmental program that adjusts plant architecture and phenology. More severe heat triggers heat-stress responses and thermotolerance mechanisms, which protect cellular function but may constrain growth and reproduction [8]. When survival-oriented responses override growth-oriented development as temperatures exceed damaging thresholds, yield losses ensue. The consequences for crop production are particularly serious. A global analysis estimated that, for each 1 °C increase in global mean temperature, yields of rice, wheat, maize, and soybean may decline by 3.2%, 6.0%, 7.4%, and 3.1%, respectively [9]. Understanding how plants respond to elevated temperature is therefore important for both crop improvement and food security.

However, “high temperature” does not describe a single biological condition. Its effects depend on temperature intensity, duration, and timing, as well as on the species, genotype, organ, and developmental stage. For example, the reported heat-stress threshold during reproductive development is approximately 26 °C in wheat but around 34 °C in rice [10]. In *Arabidopsis thaliana*, moderately elevated ambient temperatures that induce thermomorphogenesis typically range from 27 to 30 °C. Temperatures of approximately 30–37 °C begin to impose heat stress. Short-term exposure above 37 °C can disrupt plant architecture and developmental transitions. Acute exposure above 40 °C can cause severe cellular damage [11]. For instance, at the thermomorphogenesis end, temperatures from 22 °C to 28 °C activate MITOGEN-ACTIVATED PROTEIN KINASE 4 (MPK4) to phosphorylate PHYTOCHROME INTERACTING FACTOR 4 (PIF4) and promote hypocotyl elongation. At 27 °C, the Mediator component Mediator25 (MED25)–PIF4/HISTONE DEACETYLASE 9 (HDA9) complex induces YUCCA8 (*YUC8*) expression and further supports hypocotyl growth [12,13]. At the heat-stress boundary, 37 °C is a standard priming temperature for acquired thermotolerance, which requires Heat shock transcription factor A2 (*HSFA2)*. Priming at 37 °C followed by exposure to 40 °C leads to runaway reactive oxygen species (ROS) accumulation and cell death in *hsfA2* mutants [14,15]. Under moderately elevated ambient temperatures, plants initiate thermomorphogenesis. This is an adaptive developmental response to warmer, nonstressful average temperatures. This process involves changes in organ elongation, leaf position, and flowering time, and involves signaling regulation mediated by hormones such as auxin and brassinosteroids [16]. Thermomorphogenesis is thought to aid heat dissipation. It may also accelerate growth and developmental progression. In essence, it reflects promoted growth rather than growth arrest. When temperatures rise further into the heat-stress range, plants shift from thermomorphogenesis to heat-stress responses. They activate protective mechanisms such as heat shock factors and heat shock proteins to maintain cellular function. However, these responses may also impair growth and reproduction [17,18]. These heat-stress responses, particularly the HSF–HSP regulatory network, are central to thermotolerance, helping plants maintain protein homeostasis and reproductive function under heat stress [8]. Thermotolerance relies heavily on heat shock factors and heat shock proteins, which are crucial for establishing basal and acquired thermotolerance, and also involves antioxidants and membrane-related heat perception mechanisms [19,20]. Although both thermomorphogenesis and thermotolerance are induced by rising temperatures, they differ fundamentally in ecological function and signaling output. Therefore, thermomorphogenesis and thermotolerance should not be understood as two ends of a single continuum of response intensity. Instead, they are two distinct strategies that plants adopt in response to warming. The former optimizes growth and resource acquisition under nonstressful warm conditions, while the latter activates cytoprotective mechanisms to sustain survival under heat stress. These two responses have often been investigated in separate molecular contexts [21,22,23], although recent evidence indicates that their signaling pathways can interact [24].

Previous studies have identified many temperature-responsive regulators and protective mechanisms, but the developmental consequences of temperature are often discussed separately for individual pathways, organs, or stress treatments. Here, we use the plant life cycle as the organizing framework for reviewing temperature responses. We first discuss established thermosensors and candidate temperature-sensing mechanisms in crops, and then examine how elevated temperature affects seed germination, vegetative architecture, flowering, and reproductive development. Throughout the review, we distinguish adaptive developmental plasticity under warm conditions from developmental impairment under damaging heat, while considering how temperature intensity, developmental stage, organ, species, and genotype shape these responses. This perspective connects molecular temperature sensing with developmental outcomes and highlights trait combinations that may help crops maintain growth, reproduction, and yield in a warming climate.

## 2. Thermosensory Molecules and Signaling Mechanisms in Plants

**Figure 1:**
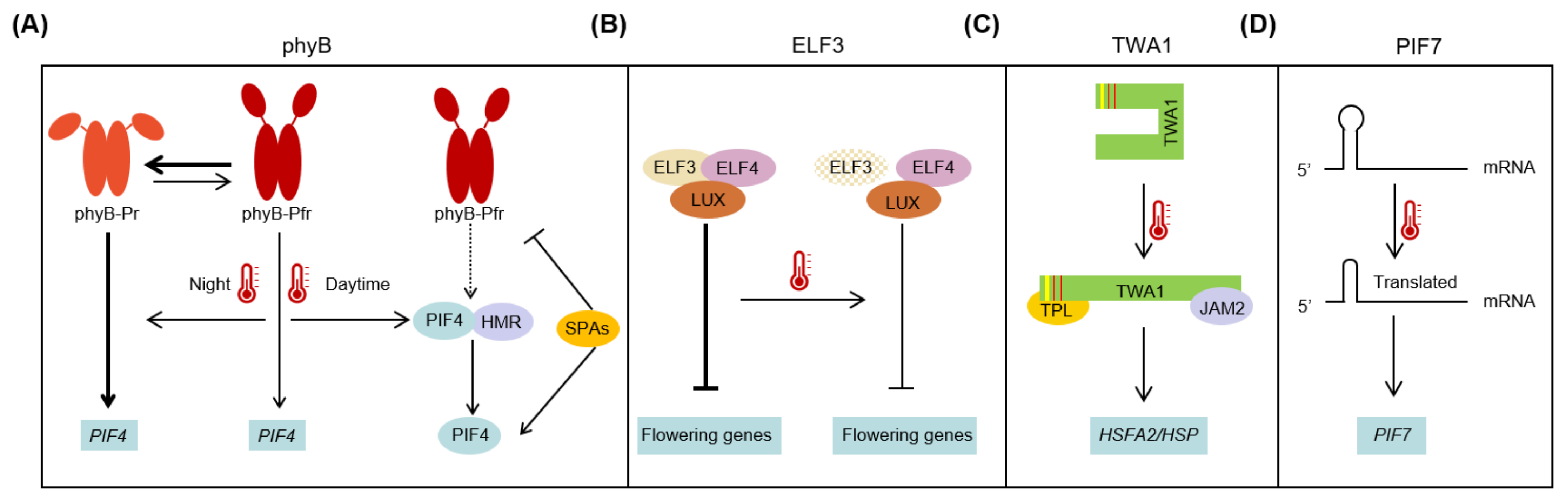
Thermosensory molecules and mechanisms. (**A**) Phytochrome B (phyB). Warm temperatures accelerate the Pfr-to-Pr conversion of phyB in darkness; as a result, the function of *PIF4* is derepressed, thereby promoting thermomorphogenesis in plants. PHYB controls daytime thermomorphogenesis mainly through the regulation of the activity and stability of PIF4 by the interaction between HMR and PIF4. Meanwhile, SPAs stabilize PIF4 and induce the destabilization of phyB at high ambient temperature. (**B**) EARLY FLOWERING 3 (ELF3). At low temperatures, ELF3 inhibits the target flowering genes in the form of the evening complex (ELF3, ELF4 and LUX). High temperatures drive ELF3 to form protein condensates, relieving the transcriptional repression on target genes, showing a phase separation mechanism of the ELF3 protein, which mediates the thermal control of flowering induction. (**C**) THERMO-WITH ABA-RESPONSE 1 (TWA1). The increased temperatures drive the conformational changes in TWA1 that allow JAM2 binding to the carboxy-terminal part of TWA1 and TPL binding to the amino-terminal domain through EAR motifs (red). The highly variable region (HVR, yellow) is integral to thermosensing. (**D**) *PIF7* mRNA conformer. At warmer temperatures, the hairpin structure within the 5’ untranslated region of *PIF7* is disrupted, leading to enhanced protein translation initiation of *PIF7*, ultimately contributing to the thermomorphogenic hypocotyl elongation. Ellipses represent proteins and the rectangle represents genes.

Plants must convert changes in temperature into molecular information before they can adjust development or activate protective responses. However, not every temperature-responsive regulator should be classified as a thermosensor. Thermosensors in plants have been identified, such as the RNA-based thermosensors, protein-based thermosensors, and plasma membrane-associated protein-based thermosensors, including photoreceptors, Ca^2+^-permeable channels, and other proteins. This review analyzes only thethermosensors that directly change their physical state in response to temperature [25,26]. Here, we distinguish direct or well-supported thermosensors, whose physical state changes with temperature, from thermosensory signaling components that transmit or interpret thermal information. Accordingly, this review considers only those factors as thermosensors whose physical state or conformation changes directly with temperature. Downstream components that relay or interpret thermal information are treated as thermosensory signaling elements, rather than as thermosensors themselves. This distinction is particularly important because thermomorphogenesis under moderately elevated ambient temperatures and heat-stress responses under damaging temperatures engage overlapping but non-identical regulatory networks (Figure 1).

### 2.1. Candidate Direct Thermosensors and Thermosensory Signaling Components in Arabidopsis

Phytochrome B (phyB) is one of the best-characterized warm-temperature sensors in *Arabidopsis*. Phytochromes interconvert between a red light-absorbing inactive form (Pr) and a far-red light-absorbing active form (Pfr) [27,28]. Warm temperatures accelerate Pfr-to-Pr thermal reversion, reduce the abundance of active phyB photobodies, and alleviate phyB-mediated repression of thermomorphogenic genes, including *PIF4* [25,29]. phyB also contributes to daytime temperature sensing through HEMERA (HMR), which promotes PIF4 accumulation and target-gene expression [30]. In addition, SUPPRESSOR OF PHYA-105 (SPA) proteins stabilize PIF4 and promote phyB degradation under elevated ambient temperatures, thereby further reinforcing the phyB-PIF4 module [31]. Although phyB homologues are widespread in crop species, direct in vivo evidence for phyB-mediated thermosensing outside *Arabidopsis* remains limited.

The evening complex (EC) provides a second thermosensory mechanism that connects temperature with circadian and developmental regulation. The EC comprises EARLY FLOWERING 3 (ELF3), ELF4, and the DNA-binding protein LUX ARRHYTHMO (LUX) and represses target-gene expression during the night [32,33,34]. At elevated ambient temperatures, ELF3 undergoes reversible condensation through its polyglutamine-rich prion-like domain, relieving EC-mediated repression of *PIF4* and promoting thermomorphogenesis [35]. ELF3 therefore represents a direct protein-based thermosensor whose temperature-dependent phase behavior is translated into developmental outputs.

THERMO-WITH ABA-RESPONSE 1 (TWA1) represents a recently identified thermosensory mechanism linked to stress tolerance. TWA1 encodes an intrinsically disordered protein whose amino- and carboxy-terminal regions adopt distinct conformations at different temperatures [36]. Thermal activation promotes its association with JASMONATE-ASSOCIATED MYC-LIKE (JAM), TOPLESS (TPL), and TOPLESS-RELATED (TPR) proteins and induces *HSFA2* and *HSP* expression. TWA1 orthologues also differ in their temperature thresholds, suggesting that variation in its biophysical properties may contribute to thermal adaptation. Given the relatively recent evidence, TWA1 is best described as a strong candidate thermosensor that connects temperature perception with ABA-related transcriptional control.

Temperature can also be sensed through RNA structure. The hairpin structure within the 5′ untranslated region of *PIF7* mRNA undergoes temperature-dependent conformational changes that increase PIF7 translation at warm temperatures [37,38]. PIF7 subsequently activates thermoresponsive genes either independently or together with PIF4, promoting thermomorphogenic hypocotyl elongation [37,39]. This RNA thermoswitch illustrates that plants can directly convert thermal information into changes in protein synthesis without requiring a protein-based sensor.

These four cases operate at distinct subcellular sites. phyB-Pfr thermal reversion, ELF3 condensation, and TWA1 action occur mainly in the nucleus. In contrast, the PIF7 RNA thermoswitch senses temperature in the cytosol and then signals to the nucleus. This compartmentalization suggests that different subcellular compartments can serve as independent sites of temperature perception. Nuclear and cytosolic inputs converge on shared downstream targets, where PIF4 and PIF7 act in parallel or cooperatively.

### 2.2. Crop- and Organelle-Associated Candidate High-Temperature Sensing Modules

Studies in crop species suggest that plants may use additional mechanisms to respond to high temperature. Candidate high-temperature sensing modules in crops can be broadly divided into those associated with warm-temperature responses (thermomorphogenesis) and those acting under damaging heat (heat-stress responses). In rice, the Thermo-tolerance 3 (TT3) locus contains two antagonistic genes, *TT3.1* and *TT3.2*, which were identified by Quantitative Trait Locus (QTL) mapping [40]. Under severe heat stress (42 °C), the plasma membrane-localized TT3.1 protein moves to endosomes. There, TT3.1 promotes the ubiquitination of the cytosolic precursor of the chloroplast-targeted protein TT3.2, leading to its degradation in the vacuole. Consequently, less mature TT3.2 enters the chloroplast. This helps protect chloroplast function and improves heat tolerance in rice. TT3.1 has therefore been proposed as a potential high-temperature sensor. However, further work is needed to determine how TT3.1 directly detects temperature changes.

On the thermomorphogenesis side, candidate modules related to warm-temperature responses have been reported in several crops, although none has been validated as a direct thermosensor in vivo. In wheat, phyB homologues are present and conserved, and PHYB genes have been characterized alongside other phytochrome family members [41]. In maize, *phyB* orthologues (*phyB1* and *phyB2*) exist, and the phyB1–PIF interaction is functionally conserved, whereas ZmphyB2 has lost PIF binding. However, heat-specific sensing by maize *phyB* remains untested [42]. In barley, an exotic *ELF3* allele contributes to developmental acclimation at elevated temperatures. This suggests that *ELF3*-based temperature responsiveness may extend to crops. However, whether this reflects thermosensing sensu stricto remains unresolved [43]. *PIF7* homologues are present in crop genomes, but whether their 5′ UTRs retain the temperature-responsive hairpin found in *Arabidopsis* remains unclear, and no functional validation of the *PIF7* RNA thermoswitch has been reported outside *Arabidopsis* [44]. These candidates primarily operate in the warm-temperature range, but direct evidence for their thermosensing capacity remains limited. Future research should prioritize staple crops with distinct thermal strategies: rice for heat stress, and wheat or maize for thermomorphogenesis. To validate candidates, we must move beyond sequence comparison to direct functional assays. Specifically, in vivo RNA structure probing (e.g., DMS-MaPseq) is needed to check if *PIF7* 5′ UTRs form temperature-sensitive hairpins. Additionally, ribosome profiling should be used to verify if warming boosts translation efficiency independent of mRNA levels. Finally, reporter assays combined with mutagenesis are essential to prove that specific RNA structures directly drive thermosensing. A summary of the current evidence for crop orthologues of *Arabidopsis* thermosensors is presented in Table 1.

Under heat stress, other organelles may also act as signaling hubs. The endoplasmic reticulum (ER) detects heat-induced protein misfolding via the unfolded protein response (UPR) [45]. In rice, the transmembrane bZIP factor *OsbZIP60* is encoded by *OPAQUE3*. It acts through a conserved mechanism to activate genes for starch and protein synthesis, as well as UPR targets, thereby maintaining ER homeostasis in endosperm cells under heat stress [46]. Beyond the ER, chloroplasts and mitochondria generate heat-induced ROS that function as retrograde messengers to reprogram nuclear gene expression. For instance, in *Arabidopsis*, the heat-induced chloroplast ribosomal protein RPS1 is required for the heat-responsive activation of the master regulator HSFA2 and its target genes [47]. These retrograde signaling pathways are well-documented under damaging heat. However, it remains unclear whether these organelles harbor intrinsic thermosensors capable of directly detecting temperature changes, independently of protein misfolding or ROS accumulation [45].

Overall, phyB, ELF3, TWA1 and the *PIF7* RNA thermoswitch are the best-characterized thermosensors in *Arabidopsis*. TT3.1 in rice extends the current picture to crop species and to different developmental settings. Orthologues of these *Arabidopsis* regulators are present in wheat, maize, tomato, and other crops. However, none of them has yet been shown to function as a bona fide thermosensor. Therefore, direct evidence for thermosensing remains largely limited to *Arabidopsis*. In crops, rice TT3.1 currently stands as the most prominent candidate in the heat-stress arm. Their direct roles in temperature sensing still need to be confirmed. Nevertheless, these findings suggest that plants may use different mechanisms to respond to warm temperatures and severe heat in different tissues and at different stages of development.

## 3. Developmental Consequences of Elevated Temperature Across the Plant Life Cycle

After plants perceive a change in temperature, the response depends on the developmental stage, the affected organ, and the severity and duration of the heat treatment. Moderate warming can alter developmental timing and growth patterns, whereas more severe heat can damage cells and disrupt development. The following sections describe how elevated temperature affects seed germination, vegetative growth, flowering, and reproductive development.

### 3.1. High Temperatures Constrain Seed Germination

Seed germination is highly sensitive to temperature and is important for seedling establishment and crop yield. When temperature rises above the optimal range for germination, it can delay or inhibit this process. The severity of this effect varies with genotype, seed condition, and the temperature treatment. For example, increasing the temperature from 21 °C to 32 °C reduced *Arabidopsis* germination by 40%, whereas rice germination decreased from 48% at 25 °C to 14% at 30 °C [48,49].

Abscisic acid (ABA) and Gibberellic acid (GA) are the main hormonal regulators of seed germination. ABA maintains dormancy, whereas GA promotes germination. Under high temperatures, ABA accumulates through the increased expression of 9-CIS EPOXYCAROTENOID DIOXYGENASE (*NCED*) genes, which encode key ABA biosynthetic enzymes [49,50,51]. Several ABA- and GA-related mutants, including *aba1*, *aba2*, *ga1–ga5*, and *ga2ox*, show altered germination because of changes in hormone content [52,53,54,55]. ABA signaling components also contribute to this response. ABSCISIC ACID INSENSITIVE 3 (ABI3), ABI4, and ABI5 promote seed dormancy, and DELLA proteins reinforce ABA signaling under unfavorable conditions. For example, the DELLA protein RGA-LIKE2 (RGL2) activates *ABI5* expression and suppresses germination [56,57].

High temperature can inhibit germination through the ABI3–ABI5–DELLA complex and its downstream target SOMNUS (*SOM*). This complex induces the expression of *SOM* and other heat-responsive genes in imbibed seeds [58]. Under high temperature, SOM interacts with AGAMOUS-LIKE 67 (AGL67) and EARLY BOLTING IN SHORT DAY (EBS). This interaction promotes SOM expression and alters ABA and GA metabolism [59]. The chromatin regulator Powerdress (PWR) acts in the opposite direction under non-stress conditions. PWR suppresses *SOM* expression by promoting histone H3 deacetylation and H2A.Z deposition at the *SOM* locus. High temperature reduces *PWR* expression and thereby releases this repression, resulting in thermoinhibition of seed germination [60].

Other signals can counteract ABA-dependent inhibition. Hydrogen sulfide (H2S) improves seed germination under high temperature by affecting the COP1–HY5–ABI5 pathway. High temperature promotes the movement of CONSTITUTIVE PHOTOMORPHOGENIC 1 (COP1) from the nucleus to the cytoplasm, allowing LONG HYPOCOTYL 5 (HY5) to accumulate in the nucleus and activate *ABI5* expression. H2S retains COP1 in the nucleus, promotes HY5 degradation, reduces *ABI5* expression, and improves seed thermotolerance [61].

**Figure 2:**
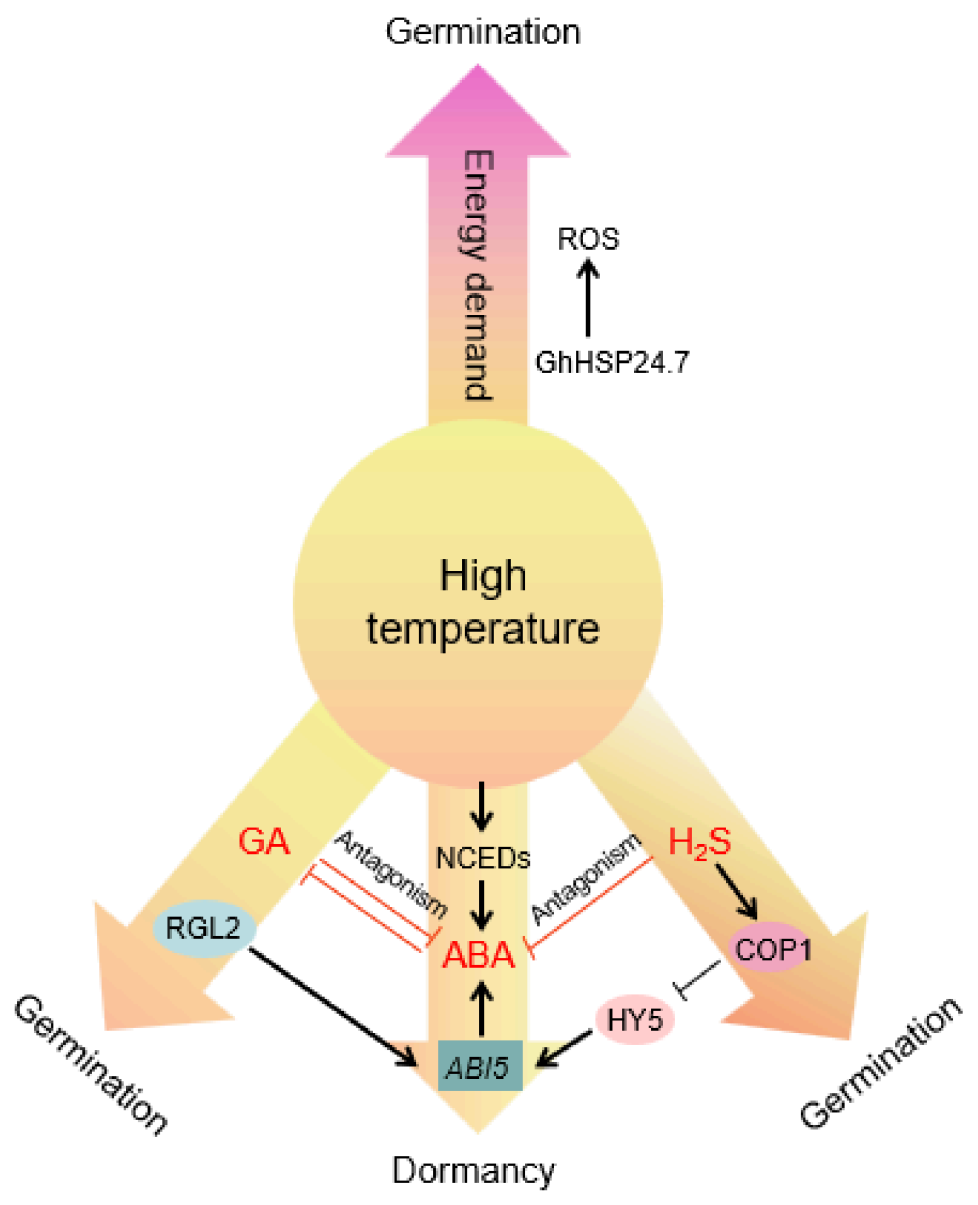
Molecular regulation of seed germination under high temperature. ABA and GA are two antagonistic phytohormones involved in seed germination, in which ABA maintains seed dormancy, while GA promotes seed germination. High temperatures induce the expressions of 9 NCEDs and cause ABA accumulation. The DELLA-type transcriptional repressor RGL2 binds to the *ABI5* promoter to induce *ABI5* expression, thus promoting ABA biosynthesis. Meanwhile, rising temperatures accelerate the COP1 efflux from nucleus to cytosol, leading to an increased accumulation of HY5 in the nuclear, which in turn promote the expression of *ABI5* and suppress seed germination. H_2_S signal could reverse the inhibition by stabilizing COP1 in the nuclear at high temperatures. In addition, multiple genes regulate seed germination via the pathways independent of the ABA and GA signals. For instance, seed germination is an energy-demanding process. A cotton mitochondrial localized protein GhHSP24.7 responds to the rising temperature, and alters the physical forces that bind seed germination by generating ROS to activate seed germination. Ellipses represent proteins and the rectangle represents genes.

Temperature also affects germination through hormone-independent pathways. In cotton, the mitochondrion-localized small heat shock protein GhHSP24.7 regulates germination in response to elevated temperature [62]. GhHSP24.7 competes with the cytochrome c maturation protein GhCcmFc and affects cytochrome C/C1 production and reactive oxygen species accumulation. These changes promote endosperm rupture and germination. This finding suggests that mitochondrial activity and redox signaling can also contribute to the temperature response of germinating seeds (Figure 2).

### 3.2. Vegetative Growth and Plant Architecture Under High Temperatures

**Figure 3:**
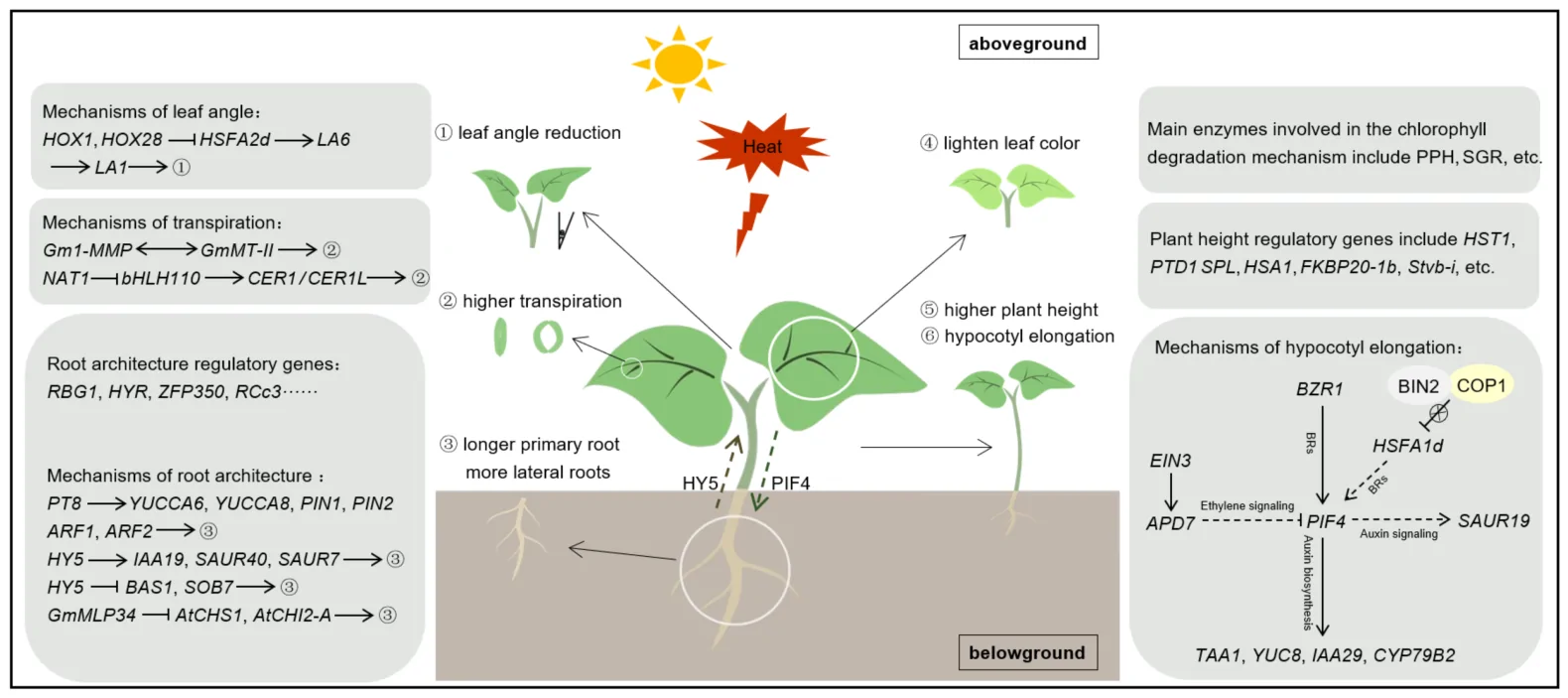
The mechanisms of plant architectural changes induced by high temperature. Increased ambient temperature triggers structural changes in both aboveground and belowground organs. Adaptation of the aboveground parts mainly included changes in plant height, leaf color, petiole angle, and stomatal opening, while the response of the belowground parts included changes in the length of the primary root and the number of lateral roots. The interaction between shoot and root systems under heat stress further highlights the coordinated regulation of plant structures in response to environmental warming.

After germination, elevated temperatures can reshape vegetative growth and plant architecture. In *Arabidopsis*, warm conditions promote the elongation of stems, hypocotyls, and petioles and alter leaf angle [63,64]. These changes may raise leaves above the soil surface and help plants avoid heat radiating from the ground. In crops, however, changes in plant height, leaf size, leaf angle, and root architecture can also affect lodging, canopy water use, light interception, and ultimately yield. It is therefore important to distinguish adaptive architectural responses to warm temperatures from reduced growth caused by heat injury. The following sections discuss how high temperatures affect shoots, leaves, and roots during vegetative development (Figure 3).

#### 3.2.1. Stem and Hypocotyl Growth

Stem elongation is highly sensitive to temperature. In maize, a 7-day heat treatment promoted internode growth before flowering, but the effect became weaker when heat was applied at a later stage [65]. Elevated temperatures also increased plant height in soybean [66]. These results suggest that the effect of heat on stem growth depends on both temperature and developmental timing. Increased elongation may help plants adjust their canopy under warm conditions, but excessive internode growth can increase lodging risk.

It is important to distinguish adaptive stem elongation from reduced plant height caused by heat injury. Rice mutants affecting *OsHST1*, *Stvb-i*, *PTD1*, *SPL7*, *HSA1*, or *FKBP20-1b* show reduced height under heat stress [67,68,69,70]. In these cases, shorter plants are more likely to result from impaired meristem function, membrane stability, or general heat tolerance than from defects in a specific heat-induced stem-elongation pathway. For example, *OsHST1* encodes a β-ketoacyl carrier protein reductase, and its loss disrupts fatty acid biosynthesis and membrane stability under heat stress [67]. Similarly, *Stvb-i* helps maintain meristem growth under high temperature while also conferring resistance to rice stripe virus [69].

Hypocotyl elongation in *Arabidopsis* is one of the best-characterized examples of thermomorphogenesis. When the temperature increases from 22 °C to 28 °C, phyB thermal reversion and ELF3 phase separation relieve repression of *PIF4*. Warm temperatures also increase *PIF7* mRNA translation [29,33,37,39]. PIF4 promotes auxin biosynthesis by activating genes such as TRYPTOPHAN AMINOTRANSFERASE OF ARABIDOPSIS 1 (*TAA1*), *YUC8*, and *CYP79B2*. It also activates *LNG1*, *LNG2*, and several SMALL AUXIN-UP RNA (*SAUR*) genes, which promote cell expansion and hypocotyl elongation [64,71,72].

Brassinosteroid signaling further promotes warm-temperature hypocotyl elongation. PIF4 interacts with BRASSINAZOLE RESISTANT1 (BZR1), and the two proteins regulate common target genes involved in cell elongation [73]. High temperature also promotes the accumulation of BZR1 in the nucleus, where it activates *PIF4* expression and forms a positive regulatory loop [74]. COP1, BRASSINOSTEROID-INSENSITIVE 2 (BIN2), and HSFA1d provide another connection between brassinosteroid signaling and thermomorphogenesis [75]. In contrast, ethylene can limit excessive elongation. Ethylene-activated EIN3 induces *APD7* expression and reduces the auxin response in the PIF4 pathway, thereby slowing hypocotyl elongation under warm conditions [76].

#### 3.2.2. Leaf Traits and Canopy Architecture

Leaves are directly exposed to solar radiation and play an important role in heat avoidance and cooling. Under elevated temperatures, plants may produce smaller or fewer leaves, alter leaf angle, or change stomatal traits [63,77]. These responses can reduce heat load or improve leaf cooling, but they can also reduce photosynthesis or increase water loss.

In *Arabidopsis*, warm temperatures promote hyponastic growth, which raises leaf position through faster growth on the abaxial side of the petiole [71]. In rice, tiller angle is controlled by auxin distribution and can respond to heat-related signals. LAZY1 (LA1) is a key regulator of rice tiller angle [78,79]. Short-term high temperature increases the expression of HEAT SHOCK FACTOR A2D (HSFA2D), which affects tiller development through the HSFA2D–LAZY-associated pathway. This pathway changes auxin distribution and influences shoot gravitropism and tiller angle [80,81]. OsHOX1 and OsHOX28 also regulate tiller angle by suppressing *HSFA2D* expression [82].

Stomatal opening can cool leaves through transpiration, but this benefit comes at the cost of water loss. In *Arabidopsis*, overexpression of *Gm1-MMP* altered leaf anatomy and stomatal traits and was associated with improved heat tolerance [83]. The transgenic plants showed changes in palisade and spongy mesophyll cells as well as increased stomatal aperture and density. These changes may improve leaf cooling, although excessive transpiration can lead to water loss and wilting. In rice, the NAT1–bHLH110–CER1/CER1L pathway promotes wax deposition in leaves and reduces water loss under heat stress [84]. Together, these studies show that leaf cooling and water conservation must be balanced, especially when heat occurs together with drought.

Heat-induced leaf yellowing and stay-green traits are not strictly architectural responses, but they affect how long the canopy remains photosynthetically active. In cool-season grasses, high temperature can accelerate chlorophyll degradation and leaf senescence [85]. SGR-mediated chlorophyll degradation can help maintain photosystem stability under heat stress, whereas stay-green genotypes can maintain photosynthesis and grain filling when functional leaf area is retained [86,87,88,89,90]. Thus, the value of a stay-green phenotype depends on whether delayed senescence is accompanied by sustained photosynthetic function.

#### 3.2.3. Root Growth and Coordination with Shoot Responses

Root systems are also highly responsive to temperature. Under moderate warming, primary-root growth and lateral-root formation can change, whereas severe heat often inhibits root growth and reduces water and nutrient uptake [91,92]. In *Arabidopsis*, auxin transport and signaling play central roles in root thermomorphogenesis. Elevated temperature can increase PIN2-mediated auxin efflux and reduce the abundance of the endoplasmic-reticulum-localized intracellular auxin transporter PILS6, leading to greater nuclear auxin accumulation and root elongation [93,94].

HY5 is another important regulator of root thermomorphogenesis in *Arabidopsis*. HY5 promotes the expression of auxin-related genes, including *IAA19*, *SAUR40*, and *SAUR77*, in roots [95]. High temperature also affects brassinosteroid signaling in roots. HY5 directly activates *BAS1* and *SOB7*, whereas HY5 degradation at high temperature reduces their expression and increases the abundance of active brassinosteroids. This response promotes root elongation [95]. However, another study showed that elevated temperature reduces the abundance of the brassinosteroid receptor BRI1, which weakens brassinosteroid signaling and also promotes root elongation [96]. These results indicate that the role of brassinosteroids in root growth depends on the temperature treatment and developmental context in *Arabidopsis*. Two speculative explanations can be considered. One possibility is that BRI1 may exert a restraining effect on a growth-promoting pathway, even without strong canonical BR signaling. In this scenario, BRI1 downregulation at warm temperatures would relieve this restraint. This would promote root elongation, without requiring a proportional change in downstream BR signaling output. Alternatively, BR signaling may differentially regulate cell division in the meristem and cell elongation in the elongation zone. Under this model, reduced BRI1 would preferentially affect meristematic activity, while cell expansion remains less affected. Consequently, the net increase in root length would reflect a shift in the balance between zones, rather than a uniform change in elongation rate. Discriminating between these possibilities would require measuring BRI1 abundance and BR signaling output separately in meristematic and elongating root zones under matched temperature regimes.

Several crop studies link root architecture with heat tolerance. In tobacco, overexpression of rice gene *OsPT8* increased the expression of auxin-related genes, including *NtYUCCA6*, *NtYUCCA8*, *NtPIN1*, *NtPIN2*, *NtARF1*, and *NtARF2*. These changes were associated with longer primary roots, more lateral roots, and improved heat tolerance [97]. Overexpression of *OsRBG1*, *OsHYR*, *OsZFP350*, or *OsRCc3* also increased primary-root length and lateral-root number in rice under stress conditions [98,99]. By contrast, high temperatures inhibit root growth in tomato and reduce water and mineral uptake [100]. In *Arabidopsis*, overexpression of soybean gene *GmMLP34* reduced root viability, primary-root length, lateral-root number, and antioxidant capacity under heat stress [101]. Thus, a larger root system is not always associated with greater heat tolerance; the outcome depends on species, temperature regime, and the balance between growth and stress protection.

Shoot and root responses share several regulators, but these regulators can have different roles in different organs. In shoots, the PIF4–SPA module is important for hypocotyl elongation at warm temperatures. In roots, the HY5–SPA module contributes to root thermomorphogenesis in *Arabidopsis* [31,95]. PIFs, HY5, and SPA proteins also affect hypocotyl thickness in response to elevated temperature [95]. In *hy5* mutants of *Arabidopsis*, both shoot and root thermomorphogenic phenotypes can be partly rescued by reducing PIF expression, suggesting that HY5 and PIFs act together to regulate temperature-responsive growth in different tissues [102]. The DET1–COP1–HY5 pathway also regulates *PIF4* expression under elevated ambient temperature in *Arabidopsis* and provides another connection between light signaling and thermomorphogenesis [103]. These findings support coordinated regulation of shoot and root growth, although they do not yet demonstrate a defined mobile thermosignal between the two organs.

#### 3.2.4. DNA Methylation, Histone Modifications, and Small RNAs in Temperature Responses

Additional epigenetic layers are also involved in shaping plant temperature responses. For instance, non-CG DNA methylation is maintained by DOMAINS REARRANGED METHYLTRANSFERASE 2 (DRM2) and CHROMOMETHYLASE 3 (CMT3). This modification represses SUPPRESSOR OF drm1 drm2 cmt3 (*SDC*) and thereby modulates thermomorphogenic hypocotyl elongation in *Arabidopsis* [104]. Under heat stress, DNA methylation and the RNA-directed DNA methylation (RdDM) machinery maintain the silencing of the heat-activated *ONSEN* retrotransposon. In methylation-deficient plants, *ONSEN* is transcribed. After prolonged heating, it can even undergo transgenerational transposition [105]. Histone modifications provide a further layer of regulation. For example, the HISTONE DEACETYLASE 9 (HDA9) – POWERDRESS (PWR) complex mediates histone deacetylation at *PIF4* target loci such as *YUC8*. This facilitates H2A.Z depletion and gene activation [106]. In addition, the H3K27 demethylase RELATIVE OF EARLY FLOWERING 6 (REF6) cooperates with PIF4 to activate thermoresponsive genes [107]. Furthermore, GCN5-mediated H3K9 and H3K14 acetylation promotes the activation of heat-shock genes, including *HSFA3* and UV-HYPERSENSITIVE 6 (*UVH6*), thereby contributing to thermotolerance [108]. Small RNAs add another layer: the microRNA156 (miR156)/SQUAMOSA PROMOTER-BINDING-PROTEIN-LIKE 9 (SPL9) module acts downstream of PIF4 to maintain auxin sensitivity and enable warm-temperature hypocotyl elongation [109]. These mechanisms are less well-established than H2A.Z-based regulation, and much of the current evidence, especially in crops, remains correlative.

### 3.3. Flowering Responses to Elevated Temperature

Flowering time is strongly affected by temperature, but elevated temperature does not always produce the same outcome. The response depends on temperature intensity, photoperiod, genotype, and species. In some cases, warm temperature promotes flowering and may allow plants to complete reproduction before later heat stress. In other cases, high temperature delays flowering or disrupts the transition to reproduction.

In *Arabidopsis*, increasing the temperature from 22 °C to 28 °C promotes early flowering by increasing the expression of FLOWERING LOCUS T (*FT*) [110,111]. PIF4 contributes to this response by activating *FT*. At higher temperatures, the H2A.Z histone variant is less abundant at the *FT* locus, which increases the accessibility of PIF4 and promotes *FT* expression [110,112,113].

However, this response is not universal. In cabbage (*Brassica rapa*), a temperature increase from 21 °C to 28 °C delays flowering. In this case, H2A.Z accumulates at the *FT* locus and reduces *FT* expression [114]. This opposing direction may reflect species-specific differences in how *H2A.Z* dynamics at *FT* respond to elevated temperature, given that *B. rapa* carries multiple *FT* copies with divergent promoters and regulatory contexts. Whether the temperature dependence of *H2A.Z* deposition versus eviction varies with the locus-specific chromatin environment remains to be directly tested. Thus, similar regulatory components can produce opposite flowering responses in different species. This comparison shows why flowering responses should not be inferred directly from studies in *Arabidopsis*.

**Figure 4:**
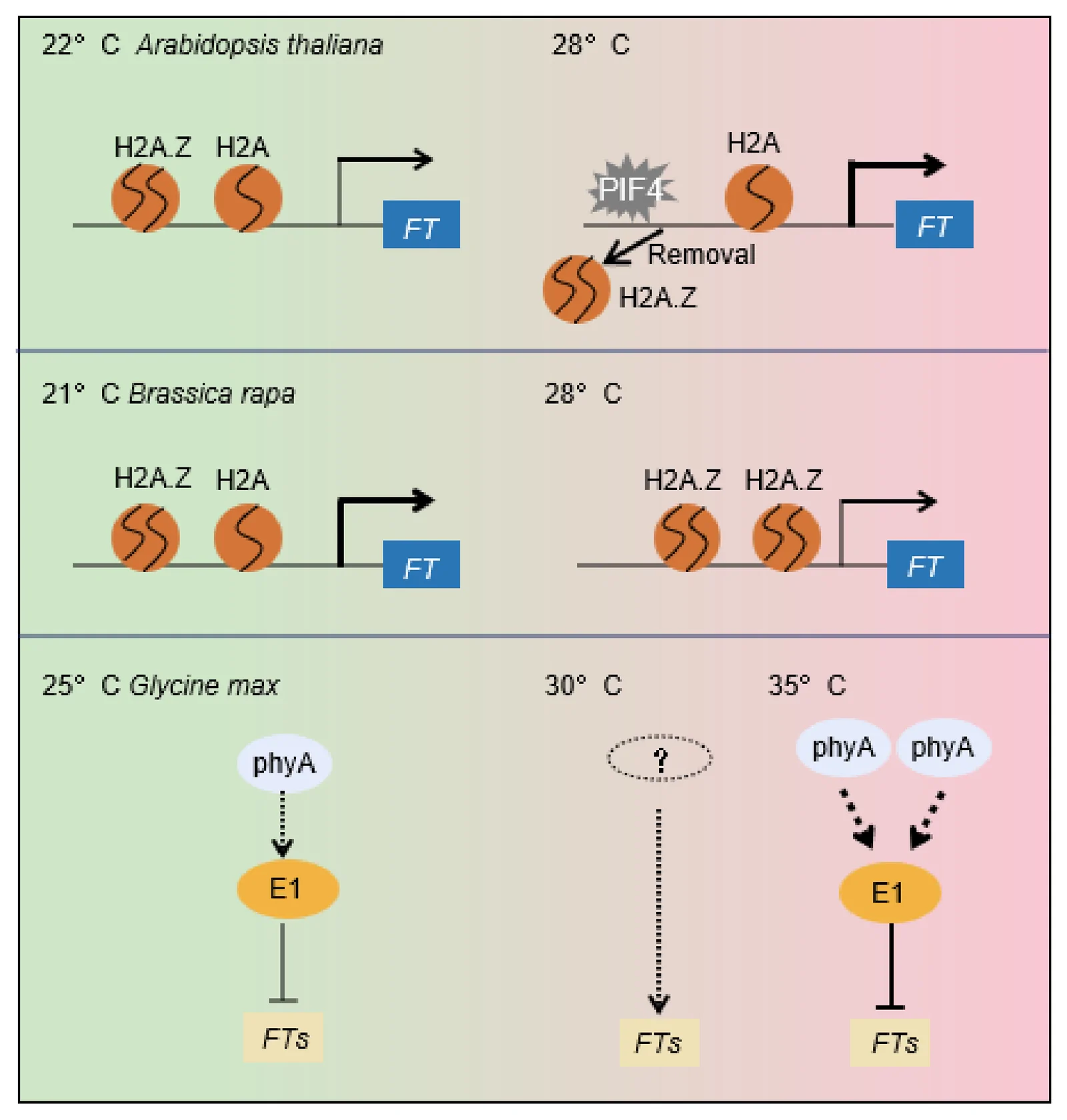
The mechanisms of different flowering responses induced by high temperature. One of the most well-known effects on plant development is the acceleration of flowering under high ambient temperatures. For example, when the temperatures rise from 22 °C to 28 °C in Arabidopsis, the occupation of H2A histone variant H2A.Z is found to be reduced at the *FT* locus, leading to the PIF4 abundance at the *FT* locus, which in turn stimulates flowering. However, contrary to that in *Arabidopsis*, a delayed flowering response when facing high temperatures is displayed because of the higher accumulation of H2A.Z at *FT* in *Brassica rapa*. In addition, rising temperatures might elicit diverse flowering responses. Up-regulation of *FT* genes induced at 30 °C leads to early flowering, which is independent of *E1*, while the photoperiodic pathway phyA-E1 is possibly crucial for 35 °C mediated late flowering. Ellipses represent proteins and the rectangle represents genes.

Soybean provides another example of the importance of temperature intensity. Under short-day conditions, increasing temperature from 25 °C to 30 °C increases the expression of *FT2a* and *FT5a* and accelerates flowering independently of *E1* [66]. At 35 °C, however, the expression of *E1*, *E1-Like a* (*E1La*), and *E1Lb* increases, probably through phyA-dependent regulation. These genes suppress *FT* expression and delay flowering [61,66,115,116]. One possible interpretation is that moderate warming directly activates *FT*. At higher temperatures, however, E1 clade repressors may become dominant and suppress *FT*. In this view, the two observations fall on opposite sides of a temperature threshold, rather than reflecting contradictory regulation. In winter barley, elevated temperature promotes flowering under long days but delays flowering under short days. Under short days, this delay is associated with the induction of the flowering repressor *HvODDSOC2* by FLC-family genes [117]. One possible interpretation is that moderate warming directly activates *FT*. At higher temperatures, however, E1 clade repressors may become dominant and suppress *FT*. In this view, the two observations fall on opposite sides of a temperature threshold, rather than reflecting contradictory regulation. Together, these studies show that flowering responses are determined by temperature together with photoperiod, genotype, and species-specific regulatory pathways (Figure 4).

### 3.4. Reproductive Development Under High Temperatures

Reproductive development is one of the most heat-sensitive stages of the plant life cycle. High temperature can affect anther development, pollen release, pollen viability, pollen-tube growth, stigma function, ovule development, fertilization, and seed set [10,118,119,120]. Damage at any one of these steps can reduce final seed production.

#### 3.4.1. Male Reproductive Development

Anther and pollen development are particularly sensitive to high temperature. Anther dehiscence is required for pollen release, but heat-induced anther indehiscence occurs in many species [121,122]. In common bean, incomplete degeneration of stomium cells contributes to this defect [123]. Studies in tomato and coffee further suggest that high temperature can alter cell-wall polymers in anthers, increasing cell-wall rigidity and preventing normal dehiscence [124,125,126].

High temperature can also disrupt pollen formation and pollen-wall development. Soybean and sorghum produce deformed pollen grains under high temperature, and the severity of pollen deformation increases as temperature rises [127]. In *Arabidopsis*, INOSITOL REQUIRING ENZYME 1 (IRE1) promotes pollen development under heat stress by splicing BASIC LEUCINE ZIPPER 60 (*bZIP60*) mRNA. The active bZIP60 protein induces genes involved in the secretion of pollen-coat materials, which are needed for normal pollen exine formation [128,129]. In *ire1* mutants, this secretory process is disrupted, leading to abnormal pollen development and male sterility under high temperature.

#### 3.4.2. Female Reproductive Development and Post-Pollination Processes

Pollen-tube growth in the pistil is essential for sperm delivery to the ovule. High temperature can inhibit pollen-tube elongation and cause pollination failure [130]. Auxin promotes pollen-tube growth in vitro, suggesting that hormone signaling contributes to pollen-tube growth in pollinated pistils [131,132]. In maize, heat stress after pollination reduces sucrose-metabolism enzyme activity and alters the expression of genes involved in sucrose degradation, trehalose biosynthesis, and hormone signaling. These changes are accompanied by increased ROS, ABA, and auxin levels and are associated with restricted pollen-tube growth [133].

High temperature also affects ovule development and function. In wheat, high temperature can increase ovule abnormalities and reduce the number of functional ovules, thereby limiting pollen-tube guidance and fertilization [134]. Cotton plants exposed to heat before anthesis produce fewer ovules and show reduced fertilization efficiency. In rice, high temperature can accelerate ovule senescence and reduce productivity [132]. These findings show that female reproductive tissues, like male tissues, are important targets of heat stress. Future studies should further examine how heat affects the later stages of fertilization, embryo development, endosperm development, and seed set.

## 4. Conclusions and Perspectives

Rising temperatures affect plant development throughout the life cycle, from seed germination and vegetative growth to flowering, reproduction, and seed set. However, plants do not respond to “high temperature” as a single, uniform signal. The developmental outcome depends on the temperature range, the duration and rate of warming, the developmental stage, the responding organ, the genotype, and other environmental conditions such as photoperiod and water availability. Moderate warming can promote thermomorphogenic growth, whereas more severe heat can disrupt cellular homeostasis and impair development. These responses are connected, but they should not be treated as the same process. Future studies should therefore define temperature treatments more carefully and consider whether a reported phenotype represents adaptive growth, heat acclimation, or growth impairment caused by heat injury.

An important question is how plants distinguish these different thermal conditions. phyB, ELF3, and the *PIF7* RNA thermoswitch provide some of the strongest evidence for direct temperature sensing in *Arabidopsis*. In contrast, TT3.1 in rice is a promising candidate whose direct thermosensory roles still require further validation. It will be important to determine the physical changes through which these proteins or RNAs respond to temperature, the temperature ranges in which they function, and the developmental contexts in which they act. Such work may help explain how plants interpret a gradual increase in ambient temperature differently from a short period of damaging heat stress.

The effects of temperature on flowering and reproduction further show why developmental context must be considered. The same temperature increase can promote flowering in one species but delay it in another, and even within a species, the outcome can change with photoperiod, genotype, or heat intensity. This is evident from the contrasting flowering responses reported in *Arabidopsis*, *Brassica*, soybean, and barley [66,117]. Similarly, reproductive heat tolerance cannot be inferred from vegetative growth alone, because a crop with an apparently healthy canopy may still suffer large yield losses when heat disrupts anther development, pollen function, fertilization, or seed set. Understanding the timing and organ specificity of these responses should therefore be a priority for future work.

Crop architecture is central to both high-yield breeding and adaptation to high temperature. The Green Revolution showed that plant height can be manipulated to improve yield: semidwarf rice varieties carrying the *sd1* allele had shorter, stronger culms and improved lodging resistance, and supported major yield gains under intensive cultivation [135,136]. High-yield and heat-resilient plant architectures therefore share a common objective—to maintain canopy productivity and grain yield while preventing lodging. However, their immediate requirements are not identical. Green Revolution breeding favored a relatively stable and compact architecture, whereas elevated temperature can promote internode elongation and increase plant height [65,137]. Moderate elongation or changes in leaf position may sometimes contribute to heat avoidance, but excessive elongation in crops can increase lodging risk and alter the allocation of resources between vegetative growth and reproduction. A heat-resilient ideotype should therefore not simply retain the shortest possible semidwarf phenotype, nor should it allow unrestricted stem elongation under high temperature. Instead, future breeding should build on elite semidwarf, high-yielding backgrounds while introducing architectural stability under heat, including moderate plant height, strong stems, controlled internode elongation, and an appropriate canopy structure. These traits should be combined with efficient water use and reproductive heat tolerance, because a suitable vegetative architecture alone cannot protect yield when heat damages floral development or seed set.

Natural variation in crop wild relatives and landraces provides an important source of alleles for this purpose. Wild soybean (*Glycine soja*), for example, is widely distributed in China and contains geographically differentiated populations that have adapted to different temperature and precipitation conditions [138]. Domestication and subsequent improvement narrowed the genetic diversity of cultivated soybean and resulted in the loss of many rare alleles present in wild soybean populations [139,140]. Wild soybean from warmer regions may therefore provide useful genetic variation for heat adaptation, but this potential should be tested directly rather than inferred from geographic origin alone. Field phenotyping, genetic mapping, and functional analysis will be needed to identify alleles that improve heat resilience without compromising yield. The same strategy can be applied to wild relatives and locally adapted germplasm in other crops.

QTL mapping, natural-allele discovery, and genome editing offer complementary routes for developing heat-resilient crops. Their value will be greatest when they are used to combine traits that act at different developmental stages, rather than to improve a single heat-response trait in isolation. For example, improved seedling establishment, thermally stable plant architecture, efficient water use, and reproductive heat tolerance may need to be combined within the same cultivar. Candidate genes and alleles should also be evaluated under fluctuating field temperatures and combined heat and drought stress, rather than only under constant conditions in controlled environments. Integrating genetic analysis with stage- and organ-specific phenotyping, transcriptomics, proteomics, and metabolomics should help identify the trait combinations that enable crops to maintain growth, reproduction, and yield in a warming climate.

## Figures and Tables

**Table 1:** Crop orthologues of *Arabidopsis* thermosensors and candidate modules.

Candidate	Crop	Evidence Type	Direct Thermosensing Demonstrated?
phyB	Wheat	Temperature-responsive co-expression network including PHY, circadian and developmental genes	No [41]
phyB	Maize	phyB1–PIF interaction reported; phyB1/phyB2 subfunctionalization	No [42]
ELF3	Barley	Developmental plasticity and yield stability at elevated temperature	No [43]
PIF7 RNA thermoswitch	Not determined	Orthologous sequences identified; structural validation lacking	No [44]
TT3.1	Rice	Temperature-dependent PM → endosome translocation (downstream event)	Proposed, not proven [40]

## Data Availability

The original contributions presented in this study are included in the article. Further inquiries can be directed to the corresponding authors.
